# Selection of the reference genes for quantitative gene expression by RT-qPCR in the desert plant *Stipagrostis pennata*

**DOI:** 10.1038/s41598-021-00833-2

**Published:** 2021-11-05

**Authors:** Rong Li, Kaiwen Cui, Quanliang Xie, Shuangquan Xie, Xifeng Chen, Lu Zhuo, Aiping Cao, Haitao Shen, Xiang Jin, Fei Wang, Hongbin Li

**Affiliations:** 1 Key Laboratory of Oasis Town and Mountain-Basin System Ecology of Xinjiang Production and Construction Corps, College of Life Sciences, Shihezi University, Shihezi, Xinjiang China; 2 Key Laboratory of Xinjiang Phytomedicine Resource and Utilization of Ministry of Education, Shihezi University, Shihezi, Xinjiang China; 3 Ministry of Education Key Laboratory for Ecology of Tropical Islands, College of Life Sciences, Hainan Normal University, Haikou, Hainan China

**Keywords:** Plant sciences, Molecular biology

## Abstract

The desert pioneer plant *Stipagrostis pennata* plays an important role in sand fixation, wind prevention, and desert ecosystem recovery. An absence of reference genes greatly limits investigations into the regulatory mechanism by which *S. pennata* adapts to adverse desert environments at the molecular and genetic levels. In this study, eight candidate reference genes were identified from rhizosheath development transcriptome data from *S. pennata*, and their expression stability in the rhizosheaths at different development stages, in a variety of plant tissues, and under drought stress was evaluated using four procedures, including geNorm, NormFinder, BestKeeper, and RefFinder. The results showed that *GAPDH* and *elF* were the most stable reference genes under drought stress and in rhizosheath development, and *ARP6* and *ALDH* were relatively stable in all plant tissues. In addition, *elF* was the most suitable reference gene for all treatments. Analysis of the consistency between the reverse transcription-quantitative PCR (RT-qPCR) and RNA sequencing data showed that the identified *elF* and *GAPDH* reference genes were stable during rhizosheath development. These results provide reliable reference genes for assuring the accuracy of RT-qPCR and offer a foundation for further investigations into the genetic responses of *S. pennata* to abiotic stress.

## Introduction

Drought is a critical environmental factor that significantly affects plant growth and crop yield. Xinjiang is an extensive arid and semi-arid region comprising the most widely distributed area of desertified land in China. The region is subject to land and wind erosion and is characterized by shifting sand dunes, which seriously hampers normal agricultural production and human development. Plants growing in this area have adapted to the extreme environment. *Stipagrostis pennata* is a desert plant distributed in the Gurbantünggüt Desert in Xinjiang that mainly grows on mobile and semi-mobile sand dunes, having important roles in wind prevention, sand fixation, and the protection of the desert ecosystem^1^. This plant has a typical rhizosheath structure around the roots, which allows the roots to resist the unfavorable external conditions of the arid desert environment and endows the plant with sand fixation ability^2^. The rhizosheaths are also the key factor by which the plants tolerate drought conditions^3^.

Reverse transcription-quantitative polymerase chain reaction (RT-qPCR) is a powerful method for detecting gene expression profiles and has been widely used in plants due to its good repeatability and high sensitivity^4–7^. The accuracy of RT-qPCR assays depends greatly on the suitability of the reference genes used^8^. Typically, the ideal reference genes should be stably expressed under different treatment conditions and in various types of cells or tissues. Many studies have shown that reference genes are not universal and that gene expression stability is relatively constant under a certain type of cell or experimental factor^7,9–11^. In plants, the candidate internal reference genes for RT-qPCR are usually housekeeping genes, including *elongation factor-1α* (*EF-1α*), *glyceraldehyde-3-phosphate dehydrogenase* (*GAPDH*), *actin* (*ACT*), *cyclophilin* (*CYP*), *phosphoglycerate kinase gene* (*PGK*), *18SrRNA*, *S-adenosylmethionine synthase* (*SAMS*), *tubulin* (*TUB*), and *ubiquitin* (*UBQ*)^12,13^. However, the stability of these genes is inconsistent under different conditions, plant species, tissues, growth and development stages, and experimental treatments^12,14–17^.

Reference gene stability has not yet been explored in *S. pennata*. This greatly limits further analysis of the functional and mechanistic elucidation of genes, thereby hindering research into the molecular basis of adaptation to abiotic stress. In the present study, transcriptome data for rhizosheath development based on RNA sequencing (RNA-Seq) were screened from the rhizosheaths of 30, 60, 90 days post-germination (DPG) plants (R30, R60, R90), and non-rhizosheath roots of 90 DPG plants (R90F). Additionally, the roots of 60 DPG plants subjected to osmotic stress were also sampled. Based on the results of three independent algorithms, eight candidate reference genes were discovered, and their expression stability was evaluated. Based on a comprehensive evaluation of their ranking, the genes of *GAPDH* and *eukaryotic translation initiation factor* (*elF*) under drought stress, *actin related protein* (*ARP6*) and *aldehyde dehydrogenase* (*ALDH*) in the plant tissues, *elF* and *GAPDH* in rhizosheath development, as well as *ARP6* and *elF* in all samples, were identified as the most stable reference genes. Our results provide reliable reference genes for RT-qPCR and further genetic function studies in *S. pennata*.

## Results

### Identification of candidate reference genes in different stages of rhizosheath development

**Figure 1:**
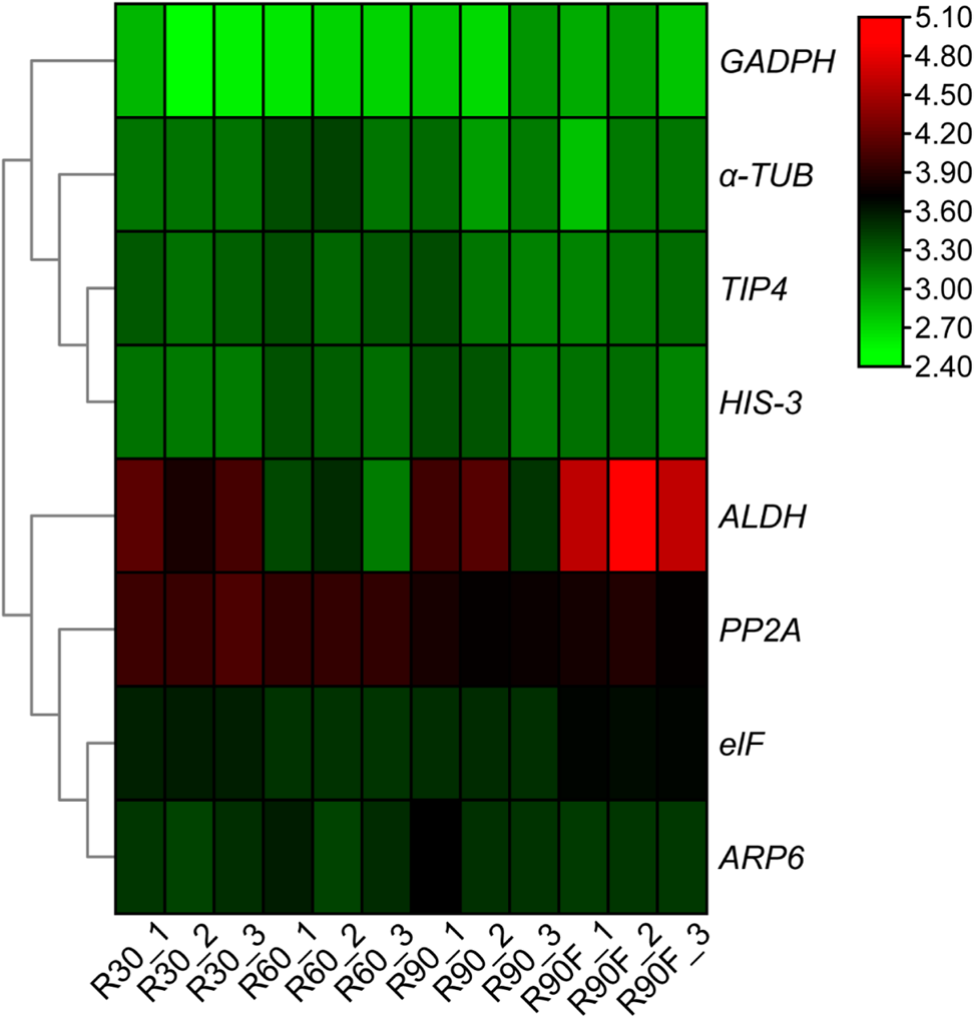
Heatmap of eight candidate reference genes based on Fragments per Kilobase of exon model per Million mapped reads (FPKM) value of transcriptome of rhizosheath development. R30, R60, R90, and R90F indicate the tissues of 30-, 60-, and 90-DPG rhizosheaths and 90-DPG rhizosheath-free roots, respectively. The screening conditions were q-value ≥ 0.05, FPKM ≥ 6, and |log_2_FoldChange| < 1. The eight candidate genes were *glycolide-3-phosphate dehydrogenase* (*GAPDH*), *aldehyde dehydrogenase* (*ALDH*), *eukaryotic translation initiation factor* (*elF*), *actin related protein* (*ARP6*), *tonoplast intrinsic protein*, (*TIP41*), *α-Tubulin* (*α-TUB*), *protein phosphotase 2A* (*PP2A*), and *Histone H3* (*HIS-3*).

The conditions of |log2FoldChange| < 1, q-value ≥ 0.05, and FPKM (Fragments per Kilobase of exon model per Million mapped reads) ≥ 6 were used to screen the RNA-Seq data, and the relatively low coefficient of variation (CV) of FPKM was set as a high standard at all sampling points, generating a total of eight candidate reference genes (Table 1). Detailed information of these genes, including unigene name, gene symbol, homologue locus, and E-value, via comparison with the homologous genes in rice is listed in Supplementary Table S1. The FPKM-based heatmap of the eight genes during different stages of rhizosheath development is provided in Fig. 1 and indicates that *GAPDH*, *α-TUB*, *TIP41*, *Histone H3* (*HIS-3*), *elF,* and *ARP6* exhibited stable expression in different stages of rhizosheath development, whereas *ALDH* and *protein phosphatase 2A* (*PP2A*) showed relative unsteady expression.

**Table 1:** Details of primers and amplification characteristics for RT-qPCR of the eight candidate reference genes.

Gene name	Primer sequence (5′–3′)	Product size (bp)	E^a^ (%)	R^2^
*GAPDH-F*	GCGTCAACGAGGACAAGTAC	151	90.9	0.994
*GAPDH-R*	GTGGCAGTGATGGAATGAAC			
*ALDH-F*	AACGGCATCCTCTGGG	127	95.3	0.995
*ALDH-R*	CCTTCACGGCTTGGTCA			
*elF-F*	CCATCCCTATGAGCCA	131	93.2	0.992
*elF-R*	ACTACTGCCAGCCTGAAGACA			
*ARP6-F*	TTCCAAGAAATGGCTCGTT	117	92.1	0.989
*ARP6-R*	TCCCATACCTCCCTCTGC			
*TIP41-F*	GGCTCAGGGTTGATGGTG	126	90.8	0.996
*TIP41-R*	TGGCAAATGTCGCTTCC			
*α-TUB-F*	TCCAGCGGCAACCTTAG	180	93.4	0.998
*α-TUB-R*	TCTCCTTCCTCCATACCTTCT			
*PP2A-F*	TGGAAATAAACAGCCAGAGC	150	95.4	0.988
*PP2A-R*	TCAATAAGTCGGATAGAACCCT			
*HIS-3-F*	CGTCGCTACCAGAAGTCG	119	92.1	0.999
*HIS-3-R*	GCACCGATGGCAGAAGA			

^a^The amplification efficiency of RT-qPCR.

### Verification of primer specificity and PCR amplification efficiency

Specific primers for the eight candidate reference genes were designed and amplified by RT-qPCR to verify the specificity using cDNA from the roots as a template. Referring to MIQE (Minimum Information for Publication of Quantitative Real-Time PCR Experiments) guidelines, we have provided information about the specific primers of the eight reference genes and RT-qPCR experiments (Supplementary Figs. S1, S2)^18^. According to the slope of the standard curve, the amplification efficiency and R^2^ of the RT-qPCR assays were calculated, which indicated that the amplification efficiency ranged from 90.8% (*TIP41*) to 95.4% (*PP2A*), and the R^2^ value ranged from 0.988 (*PP2A*) to 0.999 (*HIS-3*) (Table 1). These results indicated that all the primers of the eight genes had high specificity and amplification efficiency and were thus suitable for further analysis.

### Expression profiles of the eight candidate reference genes in *S. pennata*

**Figure 2:**
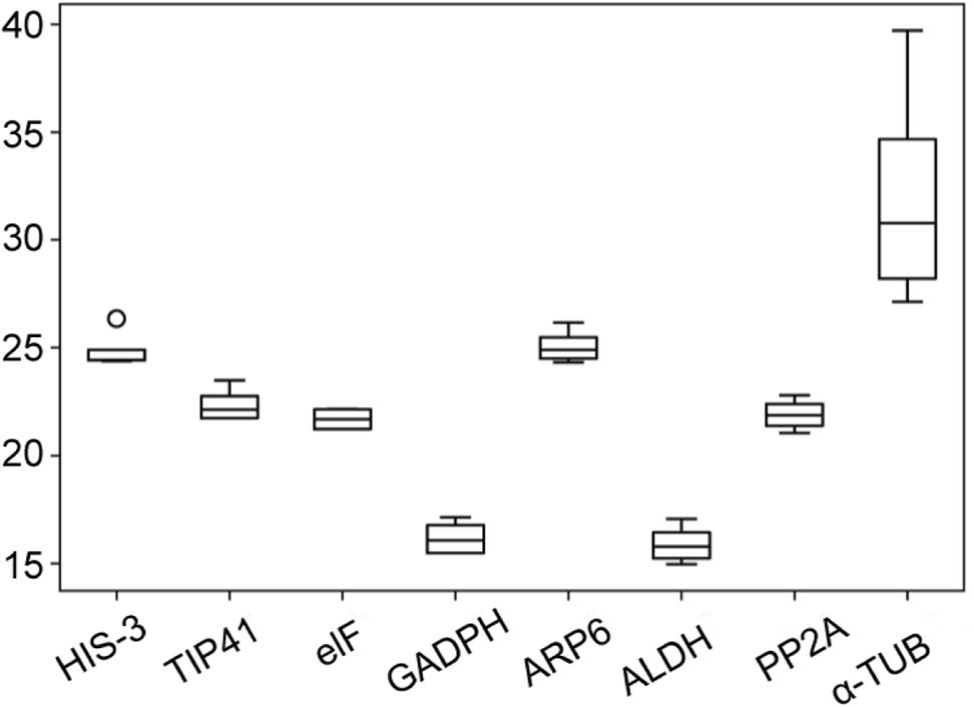
Distribution of RT-qPCR Ct values for the eight candidate reference genes across all *S. pennata* samples. The *S. pennata* materials of rhizosheaths of different development stages, roots, leaves, flowers, nodes, seeds, and PEG-treated roots were used for RNA extraction and cDNA synthesis that were then utilized for RT-qPCR reactions. Each RT-qPCR Ct value is the average of three independent experiments. The median (horizontal line), upper and lower quartiles (box), and maximum and minimum values (whisker) of each gene are displayed.

RT-qPCR assays were performed with the designed specific primers for the eight candidate reference genes using the cDNA as template extracted from different tissues and treated materials of *S. pennata*. Based on the Ct values obtained from RT-qPCR, a box diagram was generated to reflect the differences in the expression levels of the eight candidate genes (Fig. 2). The average Ct values of the reference genes ranged between 16.89 and 29.12, which indicated that the expression levels of these reference genes differed in *S. pennata*. The results showed that *ALDH* had the smallest variation, followed by *GAPDH*, *elF,* and *PP2A*, while *α-TUB* had the largest variation. Therefore, *ALDH*, *GAPDH*, *elF*, and *PP2A* were identified as relatively stable genes.

### Expression stability analysis of the eight candidate reference genes

NormFinder, geNorm, BestKeeper, and RefFinder were used to assess the expression stability of the eight candidate reference genes. NormFinder calculates the stable value (M-value) of each gene based on analysis of the variance to select the most suitable reference gene, with a low value representing high stability. The results identified *elF* and *HIS-3* for rhizosheath development with an M-value of 0.12 and 0.189, *GAPDH* and *elF* under drought stress with an M-value of 0.06 and 0.179, *ARP6* and *ALDH* in the tissues with an M-value of 0.392 and 0.58, and *ARP6* and *ALDH* in all samples with an M-value of 0.604 and 0.622 as the two most stable genes for each different treatment (Table 2).

**Table 2:** Expression stability values (M) of the eight candidate reference genes calculated by NormFinder.

Rank	Rhizosheath development	Drought	Tissues	All samples
1	*elF*	*GADPH*	*ARP6*	*ARP6*
M value	0.120	0.06	0.392	0.604
2	*HIS-3*	*elF*	*ALDH*	*ALDH*
M value	0.189	0.179	0.580	0.622
3	*GADPH*	*TIP41*	*TIP41*	*elF*
M value	0.301	0.304	0.866	0.730
4	*TIP41*	*ARP6*	*PP2A*	*PP2A*
M value	0.380	0.324	0.992	0.766
5	*PP2A*	*ALDH*	*elF*	*TIP41*
M value	0.664	0.548	1.118	0.837
6	*ARP6*	*α-TUB*	*GADPH*	*GADPH*
M value	0.796	0.588	1.719	0.991
7	*ALDH*	*PP2A*	*HIS-3*	*HIS-3*
M value	0.803	0.701	1.793	1.586
8	*α-TUB*	*HIS-3*	*α-TUB*	*α-TUB*
M value	4.187	1.817	6.184	4.245

**Figure 3:**
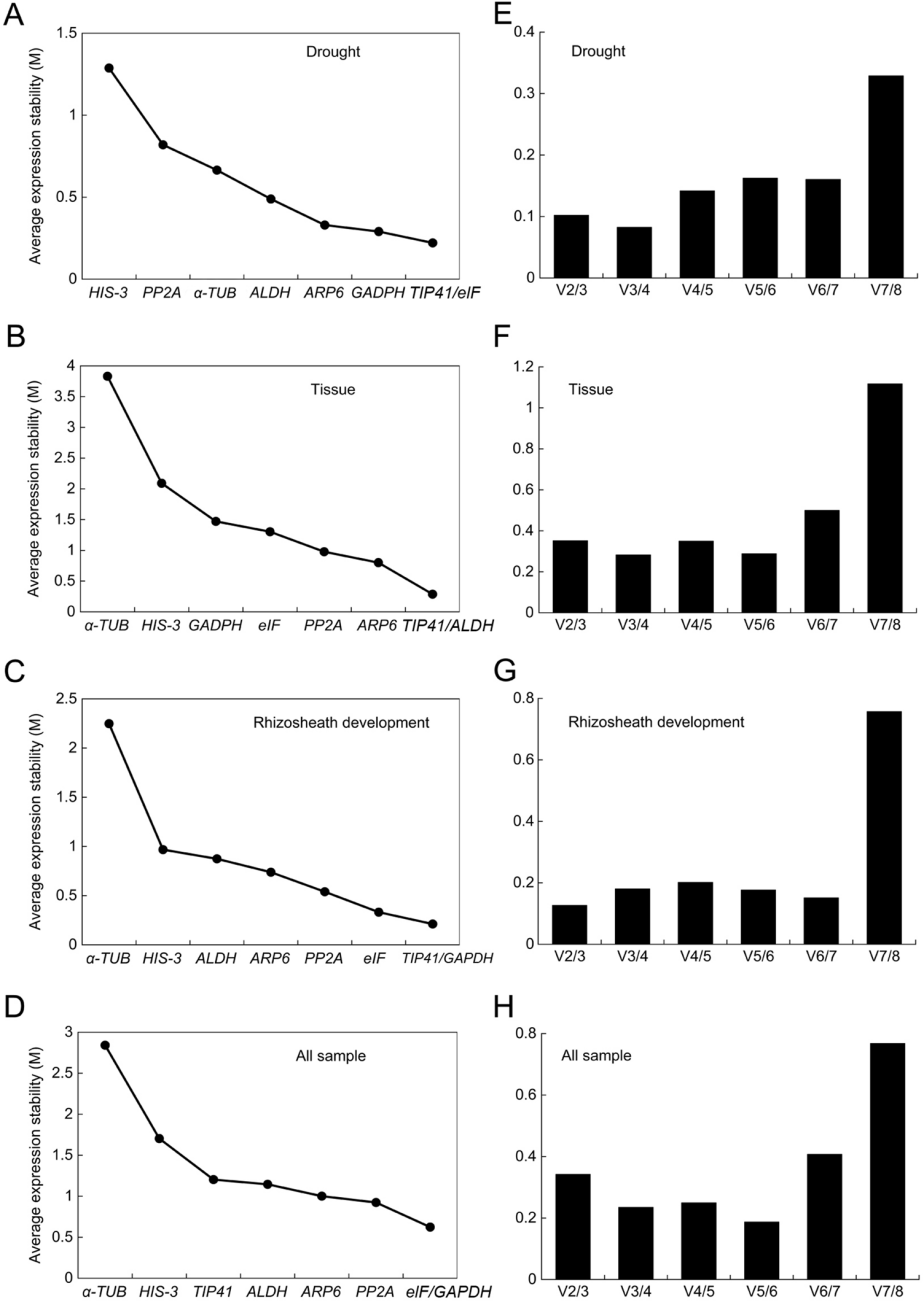
Stability ranking of the eight candidate reference genes based on M value and paired variation (V_n_/V_n+1_) value calculated by geNorm. M values represent the expression stability of each gene, with lower M value to indicate higher stability. The ratio of V_n_/V_n+1_ was used to determine the optimal reference number of multiple reference genes. M values (**A**–**D**) and V_n_/V_n+1_ values (**E**–**H**) for candidate reference genes in each group were calculated.

The geNorm program evaluates the expression stability of reference genes by calculating the M-value, with a high value representing low stability. The results showed that the M-values of the eight reference genes were all lower than 1.5 under drought stress, indicating high expression stability of these genes. In addition, *TIP41* and *elF* (M-value of 0.221) under drought conditions, *TIP41* and *ALDH* (M-value of 0.286) in all plant tissues, *TIP41* and *GAPDH* (M-value of 0.212) in rhizosheath development, and elF and *GAPDH* (M-value of 0.623) in all samples were identified as the two most stable reference genes (Fig. 3A–D). The geNorm software was also used to calculate the paired variation value V_n_/V_n+1_ (n represents the reference gene number) to determine the optimal number of reference genes for RT-qPCR standardization. The results showed that the V_n_/V_n+1_ values of the different sample groups were all lower than 0.15, suggesting that two reference genes were sufficient to complete the RT-qPCR normalization in *S. pennata* (Fig. 3E–H).

The BestKeeper procedure was utilized to analyze the expression stability of the eight candidate reference genes, with high R-values and low CV ± SD values denoting better stability^19^. As shown in Table 3, *HIS-3* in rhizosheath development (R = 0.803, CV ± SD = 2.92 ± 0.73) and *GAPDH* under drought stress (R = 0.984, CV ± SD = 6.43 ± 1.01) were identified as the most stable reference genes. *ARP6* was relatively stable in the tissues (R = 0.973, CV ± SD = 9.73 ± 2.78) and all samples (R = 0.944, CV ± SD = 6.65 ± 1.76).

**Table 3:** Expression stability values of eight candidate reference genes calculated by BestKeeper.

Rank	Rhizosheath development	Drought	Tissues	All samples
1	*HIS-3*	*GADPH*	*ARP6*	*ARP6*
R value	0.803	0.984	0.973	0.944
CV ± SD	2.92 ± 0.73	6.43 ± 1.01	9.73 ± 2.78	6.65 ± 1.76
2	*elF*	*ALDH*	*elF*	*PP2A*
R value	0.777	0.968	0.958	0.937
CV ± SD	2.11 ± 0.46	7.55 ± 1.29	12.92 ± 3.23	9.62 ± 2.00
3	*GADPH*	*elF*	*PP2A*	*elF*
R value	0.655	0.924	0.958	0.933
CV ± SD	4.36 ± 0.71	3.86 ± 0.83	12.08 ± 3.13	8.14 ± 1.86
4	*TIP411*	*ARP6*	*GADPH*	*ALDH*
Rvalue	0.48	0.918	0.918	0.931
CV ± SD	2.83 ± 0.63	4.05 ± 1.03	17.53 ± 3.54	11.17 ± 2.00
5	*α-TUB*	*TIP411*	*ALDH*	*GADPH*
R value	0.325	0.914	0.907	0.929
CV ± SD	13.25 ± 4.25	4.06 ± 0.93	10.57 ± 2.15	12.16 ± 2.12
6	*PP2A*	*α-TUB*	*TIP411*	*TIP411*
R value	− 0.293	0.811	0.858	0.905
CV ± SD	2.88 ± 0.63	2.69 ± 0.92	7.86 ± 2.00	6.37 ± 1.51
7	*ALDH*	*PP2A*	*HIS-3*	*HIS-3*
R value	− 0.336	0.591	0.791	0.858
CV ± SD	4.74 ± 0.75	3.82 ± 0.83	9.48 ± 3.03	12.38 ± 3.46
8	*ARP6*	*HIS-3*	*α-TUB*	*α-TUB*
R value	− 0.734	− 0.238	− 0.382	0.411
CV ± SD	2.54 ± 0.64	5.62 ± 1.48	14.11 ± 5.82	12.03 ± 4.34

As shown above, evaluation by these three procedures produced different results for stable reference genes from those reported in some studies^20–23^. To provide a comprehensive assessment of the eight candidate reference genes in different conditions, we performed further analysis by RefFinder^24^ according to the geometric mean of the reference genes^20,25^ to generate a final comprehensive ranking of the expression stability of the reference genes. The results showed that *elF* and *GAPDH* in rhizosheath development, *GAPDH* and *elF* under drought stress, and *ARP6* and *ALDH* in the tissues were the most stable genes. For all samples, *ARP6* and *elF* were the two most stable reference genes (Table 4).

**Table 4:** Comprehensive assessment ranking of the expression stability for the eight candidate reference genes.

Methods	Ranking							
	1	2	3	4	5	6	7	8
**Rhizosheath development**								
NormFinder	*elF*	*HIS-3*	*GADPH*	*TIP41*	*PP2A*	*ARP6*	*ALDH*	*α-TUB*
geNorm	*TIP411/GADPH*	*elF*	*PP2A*	*ARP6*	*ALDH*	*HIS-3*	*α-TUB*	
Bestkeeper	*elF*	*PP2A*	*TIP411*	*ARP6*	*GADPH*	*HIS-3*	*ALDH*	*α-TUB*
∆CT	*elF*	*GADPH*	*TIP411*	*PP2A*	*ARP6*	*HIS-3*	*ALDH*	*α-TUB*
Comprehensive assessment	*elF*	*GADPH*	*TIP411*	*PP2A*	*HIS-3*	*ARP6*	*ALDH*	*α-TUB*
**Drought**								
NormFinder	*GADPH*	*elF*	*TIP41*	*ARP6*	*ALDH*	*α-TUB*	*PP2A*	*HIS-3*
geNorm	*elF/TIP41*	*GADPH*	*ARP6*	*ALDH*	*α-TUB*	*PP2A*	*HIS-3*	
Bestkeeper	*GADPH*	*ALDH*	*elF*	*ARP6*	*TIP41*	*α-TUB*	*PP2A*	*HIS-3*
∆CT	*GADPH*	*elF*	*TIP411*	*ARP6*	*ALDH*	*α-TUB*	*PP2A*	*HIS-3*
Comprehensive assessment	*GADPH*	*elF*	*TIP41*	*ARP6*	*ALDH*	*α-TUB*	*PP2A*	*HIS-3*
**Tissues**								
NormFinder	*ARP6*	*ALDH*	*TIP41*	*PP2A*	*elF*	*GADPH*	*HIS-3*	*α-TUB*
geNorm	*TIP41/ALDH*	*ARP6*	*PP2A*	*elF*	*GADPH*	*HIS-3*	*α-TUB*	
Bestkeeper	*TIP411*	*ALDH*	*ARP6*	*HIS-3*	*PP2A*	*elF*	*GADPH*	*α-TUB*
∆CT	*ARP6*	*PP2A*	*ALDH*	*elF*	*TIP411*	*GADPH*	*HIS-3*	*α-TUB*
Comprehensive assessment	*ARP6*	*ALDH*	*TIP41*	*PP2A*	*elF*	*GADPH*	*HIS-3*	*α-TUB*
**All samples**								
NormFinder	*ARP6*	*ALDH*	*elF*	*PP2A*	*TIP41*	*GADPH*	*HIS-3*	*α-TUB*
geNorm	*elF/GADPH*	*PP2A*	*ARP6*	*ALDH*	*TIP41*	*HIS-3*	*α-TUB*	
Bestkeeper	*TIP41*	*ARP6*	*elF*	*PP2A*	*ALDH*	*GADPH*	*HIS-3*	*α-TUB*
∆CT	*ARP6*	*elF*	*PP2A*	*ALDH*	*GADPH*	*TIP41*	*HIS-3*	*α-TUB*
Comprehensive assessment	*ARP6*	*elF*	*PP2A*	*ALDH*	*GADPH*	*TIP41*	*HIS-3*	*α-TUB*

### Validation of the reference genes by RT-qPCR

**Figure 4:**
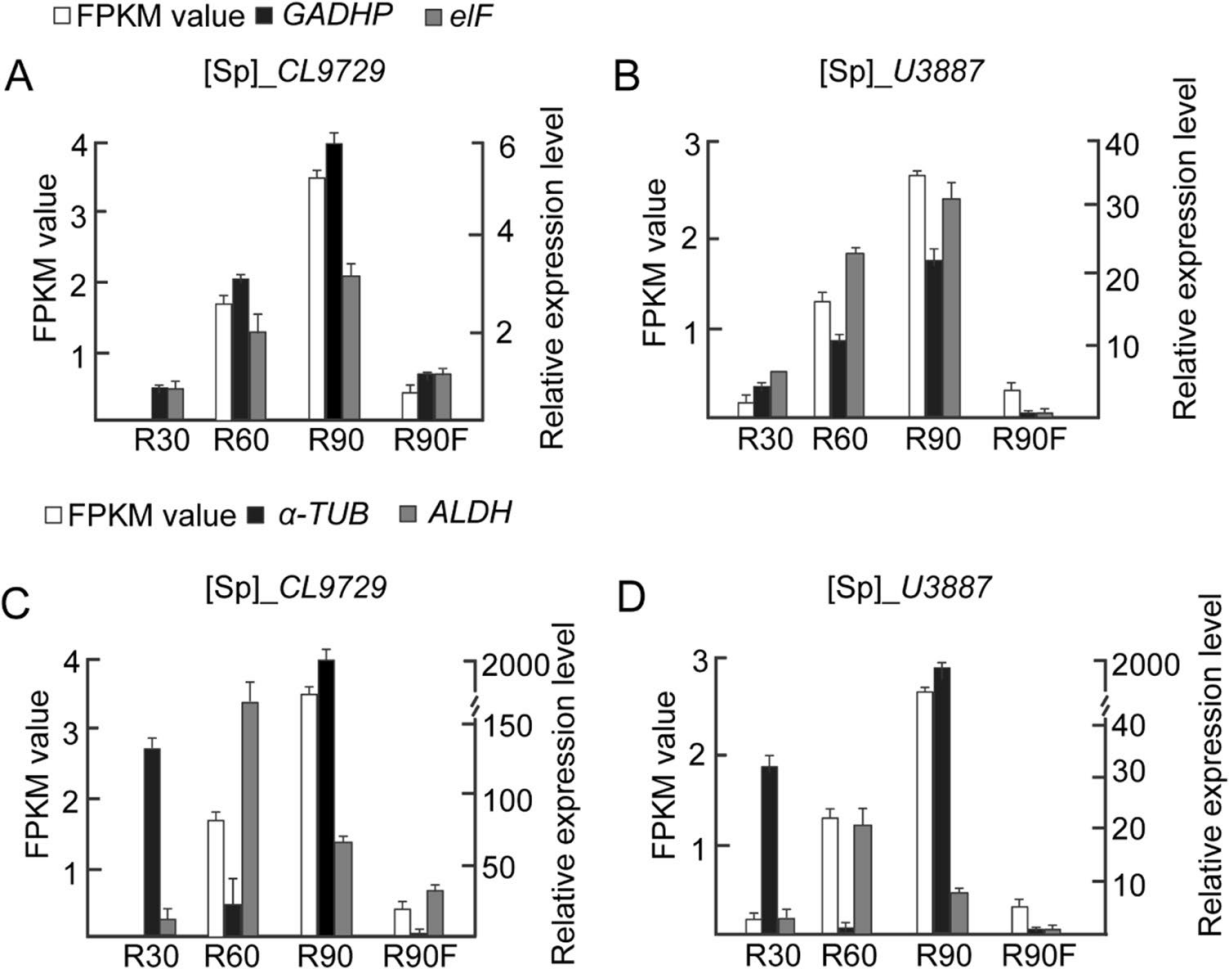
Validation of identified candidate reference genes as internal controls for normalizations of target genes of *CL9729* and *U3887*. Two stable reference genes of *GAPDH* and *elF* (A, B) and two unstable reference genes of *ALDH* and *α-TUB* (C, D) were used as internal controls to detect the expression levels of *CL9729* and *U3887* during different rhizosheath development stages. R30, R60, R90, and R90F indicate the tissues of 30-, 60-, and 90-DPG rhizosheaths and 90-DPG rhizosheath-free roots, respectively.

To verify the expression stability of the identified reference genes, two genes, namely *CL9727* and *U3887,* which are closely related to rhizosheath development, were selected for RT-qPCR to analyze the similarity between the RT-qPCR results and the RNA-Seq data. The two stable genes of *elF* and *GAPDH* and two unstable genes of *ALDH* and *α-TUB* screened by the comprehensive assessment ranking under the condition of rhizosheath development were used as reference genes for normalization to calculate the relative expression levels of *CL9727* and *U3887*. The results indicated that the expression levels of *CL9727* and *U3887* detected by RT-qPCR were highly consistent with the RNA-Seq data. When the unstable reference genes of *ALDH* and *α-TUB* were selected for standardization, the expression levels of *CL9727* and *U3887* detected by RT-qPCR were significantly different from the RNA-Seq data (Fig. 4). Additionally, correlation analysis of the relative expression levels of *CL9727* and *U3887* normalized by *elF* and *GAPDH* between RT-qPCR and RNA-Seq was performed, indicating a strong positive correlation between the RT-qPCR results and RNA-Seq data (R^2^ = 0.6061–0.9257), once again validating the stability and reliability of the two reference genes.

## Discussion and conclusion

Reverse transcription qPCR has become a common method for gene expression analysis due to its high sensitivity, good repeatability, high specificity, and high throughput, using reliable internal reference genes for the correction of target gene data to obtain accurate results^25–28^. Due to the lack of reference gene information in *S. pennata*, the reliability and accuracy of related gene expression detection significantly limits further gene function and genetic studies. Housekeeping genes such as *GAPDH* and *TUB* have typically been used as internal controls for the normalization of RT-qPCR, obtaining high effectiveness and reliability in other plant species^12,13^. However, there is great variability in the expression stability of some reference genes as a result of differences among conditions, species, tissues, growth and development stages, and experimental treatments^12,14–17^. In this study, a total of eight candidate reference genes, including *ARP6*, *elF*, *PP2A*, *ALDH*, *GAPDH*, *TIP41*, *HIS-3*, and *α-TUB,* were screened for suitability in *S. pennata* (Table 1).

The algorithms of geNorm, NormFinder, BestKeeper, and RefFinder are commonly utilized to assess the expression stability of candidate reference genes to identify the optimal reference genes, such as in *soybean*^4^, *potato*^29^, *Plukenetia volubilis L.*^30^, and *banana*^31^. Using these four programs, the eight screened reference genes showed varying expression stability under different conditions, with the different programs obtaining different results. The most stably expressed genes in the present study included *GAPDH* by NormFinder and BestKeeper under drought stress, *elF* and *TIP41* by geNorm under drought stress, *ARP6* by NormFinder and BestKeeper in all tissues, *ALDH* and *TIP41* by geNorm in all tissues, *elF* by NormFinder and geNorm in rhizosheath development, and *HIS-3* by BestKeeper in rhizosheath development (Tables 2, 3; Fig. 3). The 18S rRNA and 25S rRNA genes have been widely used as internal controls for the normalization of RT-qPCR in rice under stress conditions. In NaCl- and mannitol-treated rice seedlings, 18S rRNA and 25S rRNA showed the most stable expression levels of all reference genes^14^. It was also found that the 26S rRNA gene in *Arabidopsis thaliana* and other herbaceous plants was stably expressed under 10% PEG treatment^32^. In this study, *GAPDH* and *elF* were identified as the most stable reference genes under PEG treatment (Table 4).

**Figure 5:**
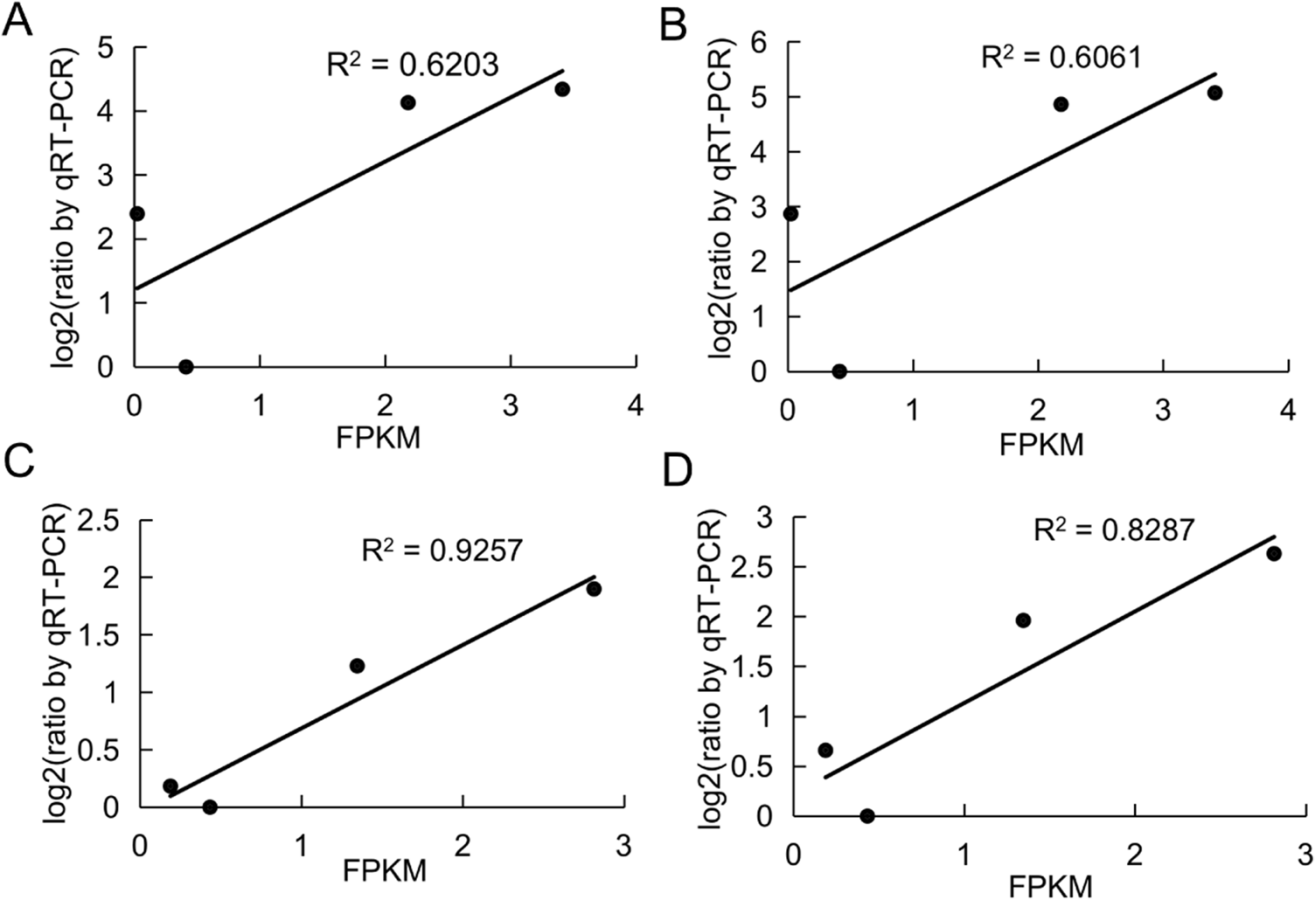
Correlation analysis of the relative expression levels of *CL9727* and *U3887* between RT-PqCR results and RNA-Seq data. X-axis represented the FPKM value in RNA-Seq data. Y-axis denoted Log_2_ (the ratio of RT-qPCR) using the method 2^−ΔΔCt^ to calculate the relative expression level. The correlation analysis between RNA-Seq data and RT-qPCR results was applied through detection of the relative expression levels of *CL9727* and *CL3887* normalized by *elF* (**A**, **B**) and *GAPDH* (**C**, **D**).

Different reference genes have been used in different plants and tissues^14,33–35^. The polyubiquitin genes *UBQ4* and *UBQ10* demonstrated the most stable expression in different tissues of *Brachypodium distachyon*^17^. *GAPDH* showed the best expression stability in different tissues and organs of *Saccharum* sp.^36^. *GAPDH* and *EF1α* exhibited better expression stability and were selected as optimal internal reference genes during fruit development in *Lycium barbarum L. EF1α* and *TUA* were used as two internal reference genes for gene expression analysis during fruit development in *Amomum villosum Lour*^37^. Our comprehensive analysis identified *ARP6* and *ALDH* in the tissues, *elF* and *GAPDH* in rhizosheath development, and *ARP6* and *elF* in all samples as suitable reference genes in *S. pennata* (Table 4). In *Vitis amurensis* Rupr. berries, the expression of *GAPDH* was more stable than in *Triticum aestivum* L.^38,39^, while *ACT* and *UBI* exhibited better expression stability in wheat but worse expression stability in *Solanum lycopersicum* L.^39,40^. Comparative analysis of the correlation between the RNA-Seq data and RT-qPCR results using the screened stable genes of elF and *GAPDH* in rhizosheath development, which was done to quantify the target gene expression of *CL9727* and *U3887,* indicated their high stability (Figs. 4 and 5), validating that these screened genes constituted reliable and effective internal reference genes for the normalization of RT-qPCR in *S. pennata*.

In conclusion, we screened eight candidate reference genes for RT-qPCR normalization based on transcriptome datasets for *S. pennata*. Using the three procedures of geNorm, NormFinder, and BestKeeper, the comprehensive assessment analysis by RefFinder, as well as comparison and correlation analysis between the RT-qPCR results and RNA-Seq data, we identified reliable and suitable internal reference genes for RT-qPCR normalization under various conditions, thereby providing a foundation for further investigations of the genetic functions and regulatory mechanisms at the molecular level in *S. pennata*.

## Materials and methods

### Plant materials

The *S. pennata* plants were collected from the area of Mosuowan Reservoir in Shihezi City, Xinjiang, and were identified by Professor Ping Yan, vice president of the Xinjiang Botanical Society and member of the plant taxonomy and phylogeny Committee of the Chinese Botanical Society. The seeds and seedlings of wild *S. pennata* are presently permitted for scientific research. A specimen was stored in the Herbarium of Shihezi University (SHI: 2018013).

The *S. pennata* seeds were grown in sand at 37 °C with a photoperiod of 18 h light and 6 h dark in a climate chamber until experimentation. The roots, nodes, and leaves of 60 DPG plants, the rhizosheaths of 30 (R30), 60 (R60), 90 (R90) DPG plants, and 90-DPG non-rhizosheaths root (R90F), as well as the flowers, mature seeds, were collected. Additionally, the roots of 60-DPG *S. pennata* plants treated with different concentrations (0%, 5%, 10%, 15%, 20%, and 30%) (w/v) of PEG 6000 (Solarbio, Beijing, China) for 36 h were also collected.

### Total RNA extraction and cDNA synthesis

The total RNA of the collected *S. pennata* materials was extracted using the RNAprep Pure Plant Kit (Tiangen, Beijing, China) according to the manufacturer’s instructions. The purity of the extracted RNA was detected by a NanoPhotometer spectrophotometer (IMPLEN, Calabasas, USA). The cDNA was synthesized from 200 ng RNA using the PrimeScript (TM) RT Reagent Kit with a gDNA Eraser (TaKaRa, Dalian, China) according to the manufacturer’s instructions.

### Selection of candidate reference genes and design of primers

Based on the *S. pennata* rhizosheath development transcriptome data obtained from DNBseq (BGI, Shenzhen, China), a total of eight reference genes were identified using the filter conditions of q-value ≥ 0.05, FPKM value ≥ 6, and |log2FoldChange| < 1. The primers used for RT-qPCR were designed according to the nucleic acid sequences by Primer (Version 5.0) software (Premier Biosoft International, Palo Alto, USA). All primers were synthesized by Sangon Biotech (Shanghai, China).

### RT-qPCR analysis

The RT-qPCR was performed on a LightCycler^®^ 480 real-time PCR system (Roche Diagnostics, Mannheim, Germany) using the SYBR Green-based PCR assay. The total reaction volume was 20 µL, which included 10 µL 2× SuperReal PreMix Plus (SYBR Green, TANGEN BIOTECH, Beijing, China), 0.5 µL each of 10 µM forward and reverse gene-specific primer, 3 µL template (first-strand cDNA), and 6 µL ddH_2_O_2_. Amplifications were performed using the following program: initial denaturation at 94 °C for 2 min, followed by a cycling procedure of 30 s denaturation at 94 °C, 30 s annealing at 55 °C, 30 s extension at 72 °C, and then a final extension at 72 °C for 10 min. The RT-qPCR analysis was tested in three biological replicates. Relative gene expression levels were calculated using the 2^−ΔΔCt^ method^41^.

### Determination and validation of the expression stability of the reference genes

The geNorm^25^, NormFinder^42^, BestKeeper^43^, and RefFinder^44^ tools are used to screen stable reference genes for data analysis based on raw quantification cycle (Cq) values. The geNorm program selects the stable reference gene by calculating the M-value of each reference gene and determines the number of optimal reference genes according to the V_n_/V_n+1_ value. The default value of V is 0.15. If V_n_/V_n+1_ < 0.15, the number of optimal reference genes is n, if V_n_/V_n+1_ > 0.15, the number of optimal internal reference genes is n + 1. The calculation principle of NormFinder is similar to that of GeNorm, and the most suitable internal parameter gene is selected according to the stability value, with the most suitable gene being the lowest stability value. The correlation coefficient (R), standard deviation (SD), and coefficient of variation (CV) of pairing between each gene can be calculated by BestKeeper, with a larger R value or smaller SD and CV values denoting better stability of the reference gene. We used the online RefFinder software (https://github.com/fulxie/RefFinder) for the comparative analysis of ΔCt. The geometric mean of the Ct values of all candidate reference genes was analyzed to rank the expression stability.

### Validation of the expression stability of the reference genes

The *CL9729* and *U3887* genes in the transcriptome data showed a close connection with rhizosheath development. In order to verify the analysis results of the candidate genes, two stably expressed (*GAPDH* and *elF*) and two unstably expressed (*ALDH* and *α-TUB*) reference genes were selected for RT-qPCR validation for these two target genes (*CL9729* and *U3887*).

## Supplementary Information

Supplementary Information 1.

Supplementary Information 2.

Supplementary Information 3.

## Abbreviations

ACT: Actin
ALDH: Aldehyde dehydrogenase
ARP: Actin related protein
Cq: Quantification cycle
CV: Coefficient of variation
CYP: Cyclophilin
DPG: Day post germination
EF-1α: Elongation factor-1α
elF: Eukaryotic translation initiation factor
FPKM: Fragments per Kilobase of exon model per Million mapped reads
GAPDH: Glyceraldehyde-3-phosphate dehydrogenase
HIS-3: Histone H3
PEG: Polyethylene glycol
PGK: Phosphoglycerate kinase gene
PP2A: Protein phosphotase 2A
RNA-Seq: RNA sequencing
RT-qPCR: Reverse transcription quantitative polymerase chain reaction
SAMSC: S-Adenosylmethionine synthase
SD: Standard deviation
TIP: Tonoplast intrinsic protein
TUB: Tubulin
UBQ: Ubiquitin
