# Supplementary figures and images for: Selection of the reference genes for quantitative gene expression by RT-qPCR in the desert plant Stipagrostis pennata
Source: Sci Rep. 2021 Nov 5;11:21711. doi: 10.1038/s41598-021-00833-2 (PMC8571334; doi:10.1038/s41598-021-00833-2)

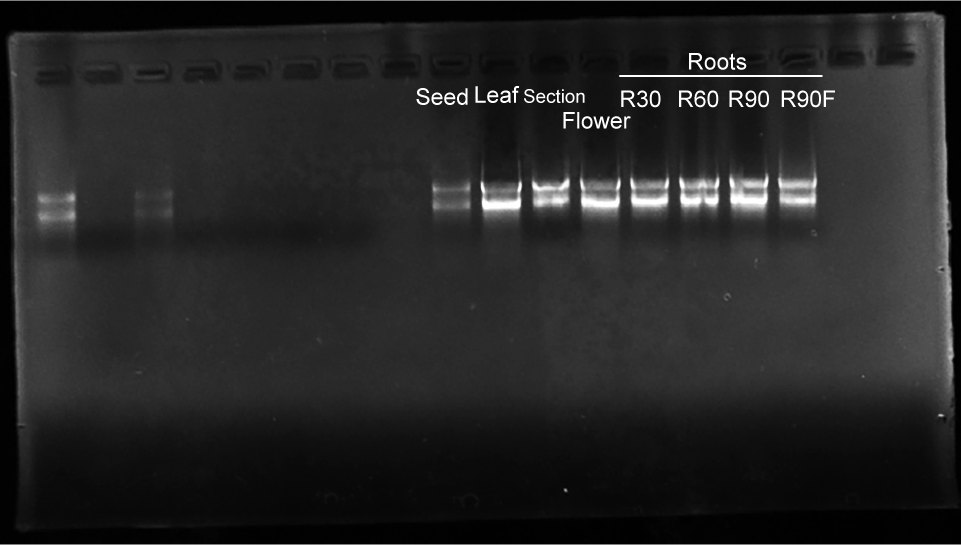

Supplement: Supplementary file 1 — Supplementary Information 1. [file 41598_2021_833_MOESM1_ESM.tif]

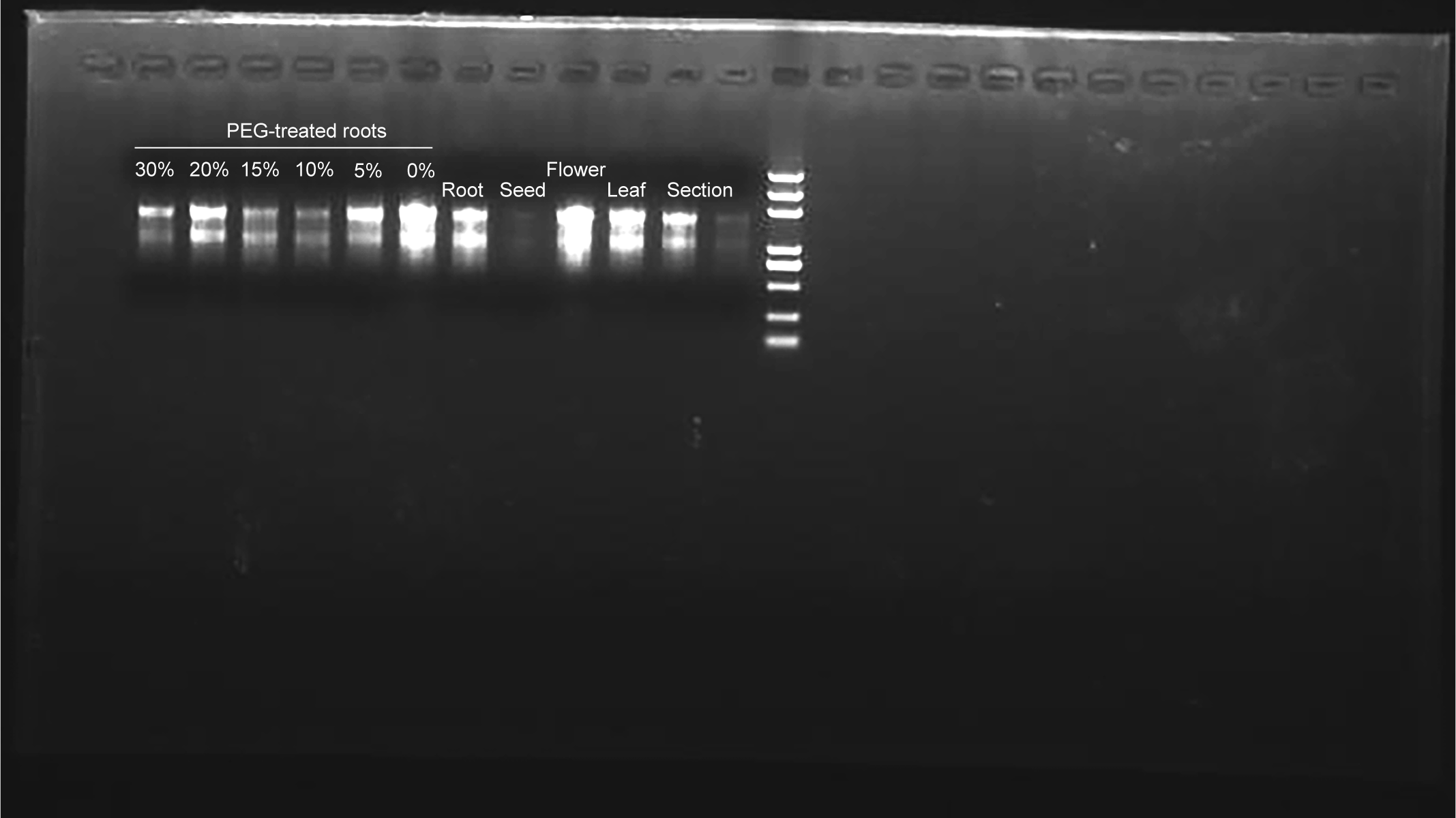

Supplement: Supplementary file 2 — Supplementary Information 2. [file 41598_2021_833_MOESM2_ESM.tif]
