# Supplementary material for: Selection of the reference genes for quantitative gene expression by RT-qPCR in the desert plant Stipagrostis pennata
Source: Sci Rep. 2021 Nov 5;11:21711. doi: 10.1038/s41598-021-00833-2 (PMC8571334; doi:10.1038/s41598-021-00833-2)
Supplement: Supplementary file 3 — Supplementary Information 3. [file 41598_2021_833_MOESM3_ESM.docx]

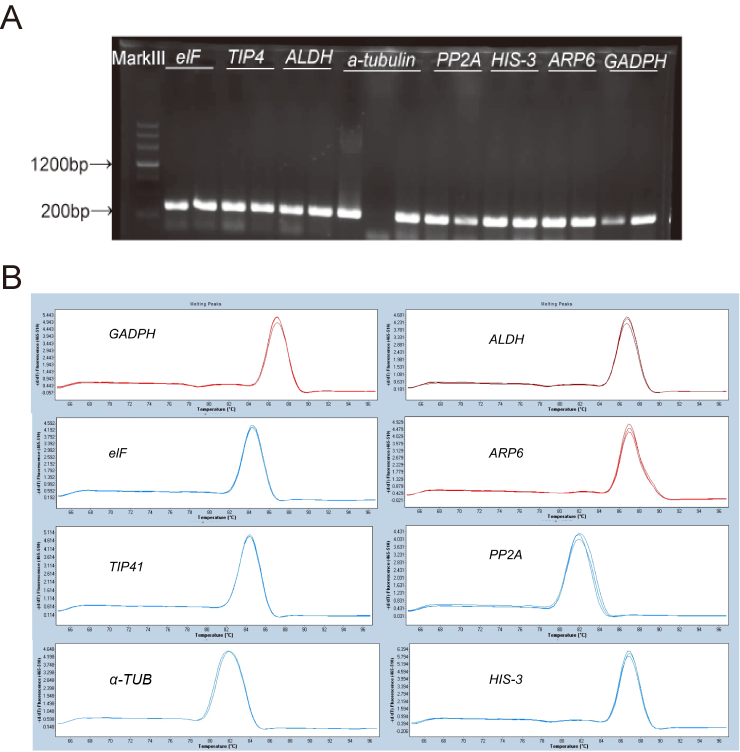


Supplementary Figure S1. Specificity identification of 8 candidate reference gene primers. A.Gel electrophoresis analysis of PCR products showed that each pair of primers produced a band. 8 candidate genes include *Glycolide-3-phosphate dehydrogenase* (*GDPH*), *Aldehyde dehydrogenase* (*ALDH*), *eukaryotic translation initiation factor* (*elF*), *actin related protein* (*ARP6*), *Tonop last intrinsic proteins, TIPs* (*TIP4*), *α-tubulin*, *protein phosphotase 2A* (*PP2A*) and *Histone H3* (*HIS-3*). B. Melting curves of the 8 selected reference genes. A unique peak was observed in each melting curve obtained by independent QRT PCR for eight candidate reference genes.


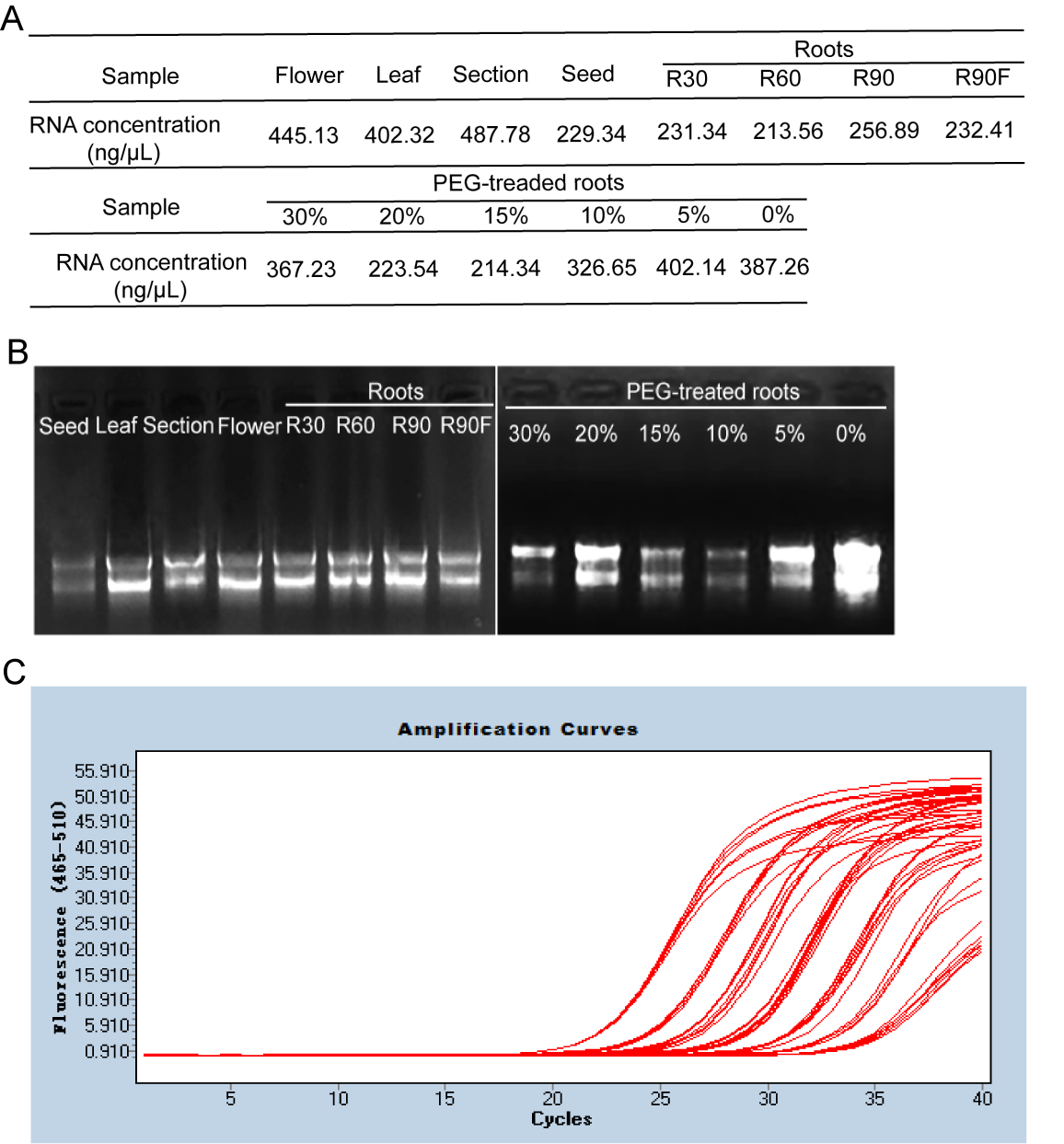


Supplementary Figure S2. Information of RT-qPCR experiment. A. RNA concentration of each sample. B. Electrophoretic map of RNA extraction results of each sample, the RNA of each sample has two bright bands without degradation. C. Amplification curve of RT-qPCR.

Supplementary Table1. Information of comparison between candidate genes and rice homologous genes.

| unigene name | gene symbol | Gene_ID | pep_ID | E value |
| --- | --- | --- | --- | --- |
| CL13580 | ALDH | DAI22_11g222300 | KAF2911992.1 | 5.12E-103 |
| CL15005 | TIP41 | DAI22_03g347200 | KAF2941447.1 | 0 |
| Unigene464 | elF | DAI22_03g377400 | KAF2941853.1 | 0 |
| Unigene45412 | GADPH | DAI22_08g167000 | KAF2919864.1 | 0 |
| CL3854 | PP2A | DAI22_06g188200 | KAF2927219.1 | 0 |
| CL1341 | ARP6 | DAI22_01g121400 | KAF2949580.1 | 0 |
| CL10782 | α-TUB | DAI22_03g322000 | KAF2941080.1 | 3.3E-113 |
| Unigene34579 | HIS-3 | DAI22_01g411400 | KAF2953481.1 | 2.61E-87 |
